# Hope in Hospitalized Patients with Terminal Cancer

**DOI:** 10.3390/ijerph16203867

**Published:** 2019-10-12

**Authors:** Bożena Baczewska, Bogusław Block, Beata Kropornicka, Antoni Niedzielski, Maria Malm, Agnieszka Zwolak, Marta Makara-Studzińska

**Affiliations:** 1 Chair of Internal Medicine and Department of Internal Medicine in Nursing, Medical University, Chodźki 7 Street, 20-093 Lublin, Poland; 2 The Pontifical University of John Paul II, Kanonicza 25, 31-002 Kraków, Poland; 3 Chair of Humanities, Medical University Staszica 4-6 Street, 20-081 Lublin, Poland; 4 Department of Medical Informatics and Statistics with E-learning Lab, Medical University, Jaczewskiego 4 Street, 20-090 Lublin, Poland; 5 Department of Health Psychology, Faculty of Health Sciences, Jagiellonian University, Collegium Medicum, Kopernika 25 Street, 31-501 Kraków, Poland

**Keywords:** hope, cancer, terminal disease, care

## Abstract

Hope is of great importance for patients diagnosed with cancer, especially those that are terminally ill. The diagnosis often puts an end to the realization of personal, social, and professional goals. The aim of this study was to characterize the hope of hospitalized patients diagnosed with cancer in the terminal phase of the disease. The research tool used in the study was Block’s hope test (NCN-36; NCN- Nadzieja Chorych Nowotworowych—Hope of Cancer Patients), designed for patients with life-threatening diseases. The results showed that the patients were characterized by a moderate level of global hope. The highest levels of hope were noted in the spiritual-religious area and the lowest levels of hope concerned curing the disease. Patients exhibited varied levels of hope and varied internal structures of hope. They presented four different types of hope: optimistic, moderate, religious, and weak. Optimistic hope was found most frequently in patients diagnosed with a terminal phase of cancer, while weak hope was represented by the smallest group of these patients.

## 1. Introduction

Hope is a fundamental quality of human nature. It is experienced regardless of age, culture, place of origin, or ethnicity. It has many meanings and is difficult to define [1]. Over the years there have been many attempts to describe it [2,3,4,5,6,7,8,9,10]. According to Snyder, hope is a dynamic and multilayered mental process, composed of three integral elements: goals, agency, and routes. It is a sum of motivational energy (internal force) and specific routes (strategies, visions, plans to realize goals) leading to the desired goal (destination). All three components are necessary for hope to work properly [11,12].

Hope among seriously ill patients is the subject of several international research studies. According to Wang and Lin [13], spiritual well-being may reduce the negative impacts of cancer on quality of life and the desire for hastened death. Thus, the study of the effectiveness of the methods leading towards mood and hope improvement in cancer patients has been undertaken [14]. Research concerning the field of hope also refer to backgrounds of medical staff enabling them to better take care of this patient group. The aim of studies performed in Norway was to explore newly qualified doctors’ learning experiences with end-of-life care in nursing homes, especially focusing on dialogues around death [15]. In turn, Canadian studies, concerning better understanding of the role of hope among terminally ill cancer patients, showed that hope changes together with disease progression. Initially, patients hope for miraculous healing, but when the therapy used is no longer effective in stopping illness progression, they enjoy the present moment and prepare for the end of life. Therefore, clinicians need to maintain some degree of hope while remaining as realistic as possible [16]. 

In Polish psychological literature, exploration of hope and its role among terminally ill cancer patients has not been yet been undertaken in this respect. The present studies were performed in order to fill this literature gap. It is hard for a patient to accept suffering and to die with dignity [17,18,19]. Therefore, the hope assessment among dying patients and their families may contribute to verifying unrealistic and illusory hope as being the source of greater life affirmation and better experiencing mourning [17,18,19]. There are two questionnaire methods to study hope available in Polish literature; however, these are not adequate for terminally ill patients. The method proposed by Łaguna et al. [20] was developed on the basis of Snyder’s cognitive–motivational conception of hope [11,21]. 

Hope is of great importance for patients diagnosed with cancer, especially when it is terminal. The diagnosis often puts an end to the realization of personal, social, and professional goals. This can lead to frustration, triggering negative emotions such as anger, sadness, anxiety, and regret. The disease disturbs the familial harmony in all spheres of its functioning.

This article will prove helpful for clinicians in treating and communicating effectively with current and future patients. Perhaps clinicians and caregivers will recognize the need for a patient to receive more spiritual or emotional support in the terminal phase of cancer. Understanding the language of hope presented in the article may assist clinicians in their conversations with patients and families. The study results provide good information and a pathway to addressing the larger existential questions that emerge in the terminal stages of cancer.

### Objectives of the Study

The aim of the study was to characterize hope in hospitalized patients with terminal cancer, in the terminal phase of the disease. We tried to answer the following questions:

- What is the intensity of hope in patients diagnosed with cancer in the terminal phase of the disease?
- What is the profile of their hope?
- What types of hope are dominant in the patients group?

## 2. Materials and Methods

The article was written according to the STROBE (The Strengthening the Reporting of Observational studies in Epidemiology) guidelines. 

In total, 246 patients in the terminal phase of cancer took part in the study. The patients were hospitalized in the palliative care and hospice care wards in 17 centers in Poland. The research group, led by doctor Bogusław Block, obtained permission from the Bioethics Committee at Medical University in Lublin (permission no KE-0254/225/2010). The study was carried out from 2010 to 2016.

The patients’ age ranged from 18 to 90, and the mean age was 59.5. The biggest group (*n* = 99) was made up of patients aged 51–65 years (40.24%), while the smallest group was made up of patients younger than 35 years of age (*n* = 14, 5.69%). Of all patients, 34.96% were older than 66, and 19.11% were between 36 and 50 years of age (*n* = 47).

The majority of patients were women (*n* = 150, 60.98%) and those living in cities (*n* = 192, 78.05%). The majority of patients lived with their family (*n* = 155, 60.01%), some patients lived with non-family members (*n* = 20, 8.13%), and the rest lived on their own (*n* = 71, 28.86%). The majority of patients were married (*n* = 117, 47.56%). The second most numerous group were widowed patients (*n* = 67, 27.24%), followed by divorced patients (*n* = 32, 13.01%) and singles (*n* = 30, 12.20%).

The majority of patients finished their education at the secondary school level (*n* = 82, 33.33%). There were 29 patients with higher education (11.79%), 59 patients with primary education (24.22%), and 59 patients with vocational education (24.22%). There were 17 patients with a bachelor’s degree (6.91%).

The patients described their financial situation as satisfactory (*n* = 111, 45.12%), bad (*n* = 73, 29.67%), good (*n* = 52, 2114%), or very good (*n* = 10, 4.07%).

The patients suffered from respiratory, nervous, digestive, urinary, osteoarticular, secretion, hemopoietic, reproductive system, and skin cancers. The patients remained anonymous throughout the study and gave consent to take part in a standardized interview. The interview with the patients was of a diagnostic and therapeutic character. The atmosphere throughout the interview was friendly, and the patients were ensured privacy. In the case of questions not being understood by the patients, the questions were clarified. 

The test used in the study was the hope test (NCN-36) developed by B.L. Block. 

### 2.1. Development of NCN-36

According to the opinion of Scioli [22] and Biller [23], there is a need to develop the hope test, reflecting hope as a multifaceted phenomenon in health and disease, including the determinants typical for a defined population. Therefore, NCN-36 (NCN- Nadzieja Chorych Nowotworowych—Hope of Cancer Patients) is the author’s test and native tool for hope assessment in cancer patients. This test is based on an empirical model derived from the background of cancer patients and is accurate for these patients who are in a situation of uncertainty and fear because of danger to life, disease progression, and many accompanying troublesome and hard to control symptoms. 

### 2.2. Development of the Test Positions

The multivariate hope scale (NCN-36) was developed on the basis of an empirical model. In the first step, 30 people caring cancer patients, such as psychologists, social workers, and volunteers, who had frequent and direct contact with terminally ill patients, were asked to give a written answer to the open question: How do you know that your patient/pupil hopes? Additionally, a literature review was done. The pool of statements was collected (*n* = 100), then they were arranged; repeated statements were eliminated and those not concerning hope were rejected by four master’s students of psychology (deemed “competent judges”). In this way, the pool of 50 statements was graded with a 7-point Likert scale, ranging from −3 to +3. The preliminary version of the test in the form of the scale consisted of 50 items with 7-point response scale. 

In the second step, the questionnaire studies were performed among 197 people taking care of terminally ill patients, who assessed their pupils hope in order to verify the discrimination power of the test positions. The obtained results were analyzed statistically by SPSS (PREDICTIVE SOLUTIONS Sp. z o.o., Kraków, Poland). Principle component analysis (PCA) with orthogonal rotation allowed extraction of four hope dimensions, named: the situational dimension (α-Cronbach = 0.84), the spiritual-religious dimension (α-Cronbach = 0.86), the affective dimension (α-Cronbach = 0.81), and the telic-temporal dimension (α-Cronbach = 0.72). Then, the positions with the highest accuracy were selected, totaling eight for each dimension. The scale contained 32 diagnostic positions and four buffer positions, totaling 36 items.

The test offers a high level of Cronbach’s alpha reliability (0.92). The results range from 1 to 7. The interpretation is based on the mean average value of the results. The results in the 6.0–7.0 range point towards a strong level of hope, 5.0–5.99 indicates a moderate level of hope, 3.0–3.99 indicates a low level of hope, 2.0–2.99 indicates a very low level of hope, and 1.0–1.99 indicates hopelessness.

The situational dimension is related to uncertain hope for recovery or health improvement, concerns about the effectiveness of the therapy, the level of trust towards doctors, and belief in the effectiveness of medicine (both scientific and nonconventional).

The telic-temporal dimension deals with hope related to the patients’ attitude towards their future. The patients have many reasons to live, since they are convinced that the future has a lot to offer. They have important goals to achieve in their lives, and they have dreams, plans, and ambitions to fulfill. This component of hope becomes an indirect motivational function of hope, which spurs the patients into action and encourages them to put a lot of effort into recovery. The patients care about their health improvement. They are motivated to cooperate with doctors more efficiently and adhere to their recommendations. 

The hope of a spiritual-religious character refers to the Supreme Being, personified God, whom the patients trust and lay their hopes on. The patients obtaining high results in this subscale are willing to trust God with their lives and their future, which are subject to Gods love and care. This will give them a spiritual support and peace of mind in difficult times. The patients with such a hope look at life from a perspective that goes beyond death, the perspective of an eschatological time. Strong spiritual hope may facilitate the acceptance of one’s life and fate, but also at the same time may lead to passivity and apathy. 

The affective component of hope makes the patients determined not to give in to anxiety but rather to fill them with courage and endurance so that when they think about the future they experience inner peace. This component of hope seems to be contrary to the feelings of sorrow, depression, and strain resulting from a difficult and uncertain future. The results in an affective dimension point towards the patients’ experiencing ambivalent states, such as anxiety about an uncertain future, suffering, and death, yet also feelings of optimism and courage. 

The scale of hope experienced by the patients is reflected by their global results. The profile of hope is shaped through the results of each particular scale.

### 2.3. Methods of Statistical Analysis

A statistical analysis of the results is presented in the tables in the percentage form, together with the mean average results and standard deviation, the minimal and maximal values, and modal and median values. Friedman’s analysis of variance (Friedman’s ANOVA) test was used to contrast the studied types of hope, together with a hierarchical cluster analysis and an analysis of variance (ANOVA I). The calculations were done with the use of STATISTICA 13.0 software (StatSoft Polska Sp. z o.o., Kraków, Poland). Statistically significant differences and dependencies were found at *p* < 0.005.

## 3. Results

The measurements of hope in hospitalized patients with cancer in a terminal phase are presented in Table 1. 

The global results show that the patients are characterized by a moderate level of hope (M = 4.52, Me = 4.47). Most patients experience varying degrees of hope, from weak to strong (3.73–5.31, SD = 0.79). The less numerous groups contained patients with a very low level of hope, or patients without hope. Some patients exhibited a very high level of hope (Max = 6.34).

There are significant differences in the intensity of hope for its particular dimensions. The internal structure of hope is presented by the mean results of its four dimensions. The dominant component of hope in the studied group is its spiritual-religious dimension (M = 5.34). The level of hope in patients with life-threatening cancer with a poor prognosis for recovery in the telic-temporal dimension is moderate (M = 4.74). Also, for the affective dimension, the level of hope is moderate (M = 4.15). The situational content is of the lowest value (M = 3.85) for the internal structure of hope.

**Figure 1:**
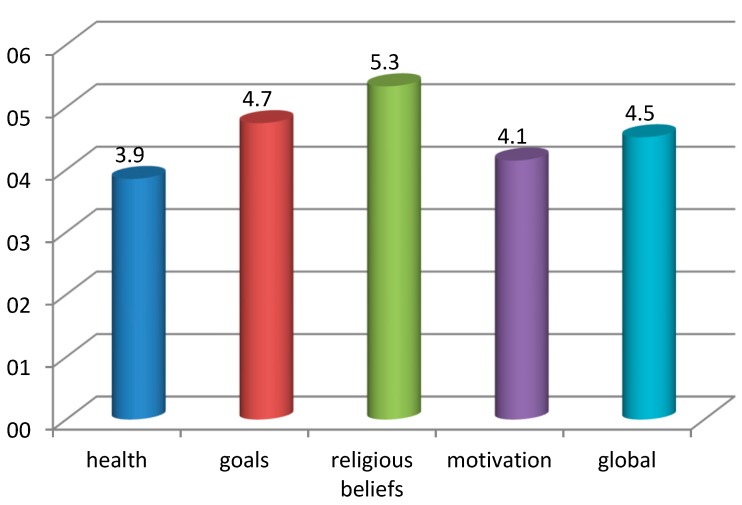
The profile of the patients’ hope in a terminal phase of cancer.

The mean results in the four scales make up the global profile of the patients’ hope. These are presented in Figure 1.

The results presented in Figure 1 show that hope in the religious dimension is the most intense, with a slightly higher value than the hope of achieving desired goals in the future. Lower on the scale is the hope expressed in an effective way and the hope for recovery. However, the patients exhibit intraindividual and interindividual differences in the internal structure of hope. The data are shown in Table 2.

As seen below, when analyzing data presented in Table 2, we included in parentheses the percentage of patients whose level of hope was within a certain range (results range column). The patients thinking about the future experience feelings of anxiety, tension, and unrest. This is confirmed by the results (37.40%; 2.0–3.99) in the affective dimension. Only a few patients (0.81%) show high levels of hope in this dimension, which can be interpreted as the acceptance of their situation. This may result from them not being fully aware that their lives are threatened. The most numerous group experiences a very high (34.15%; 6–7.00) or high (34.55%; 5–5.99) level of spiritual-religious hope. Their hope results from a deep belief in God’s help in difficult times, to whom they trust their disease and recovery. In total, 4 out of 10 patients (42.68%) exhibit strong or very strong hope in the telic-temporal aspect. This means that despite a life-threatening disease, they want to make plans and realize short-term and long-term goals. More than half of the patients (55.19%) results below 4.0 (1.00–3.99) for the situational dimension. The patients in this group do not believe in the effectiveness of the therapy, do not hope for recovery, and some of them feel depressed and hopeless.

**Figure 2:**
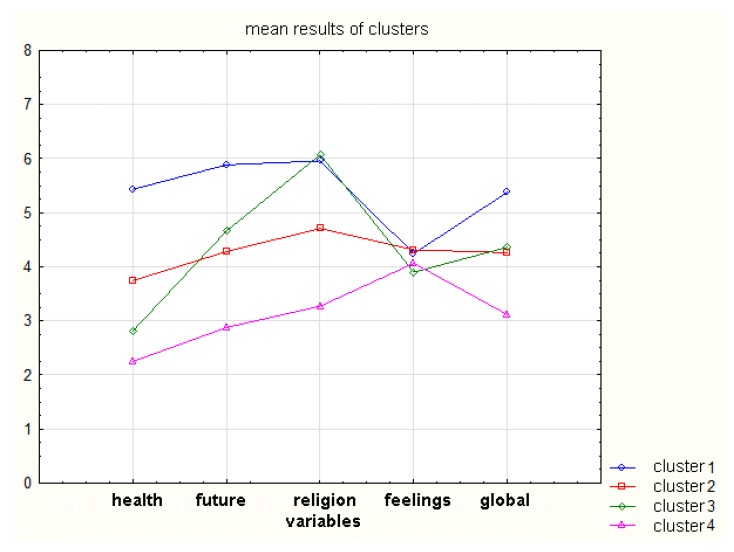
Profiles of hope types for patients in the terminal phase of cancer.

When faced with a terminal disease and a poor prognosis for recovery, patients experience various levels of hope, which is not homogenous in its structure. The profiles of hope can be distinguished by applying a hierarchical cluster analysis. The obtained results are presented in Table 3 and in Figure 2. The cluster analysis helps to differentiate between four types of hope, which show a high level of statistically significant difference (*p* ≤ 0.000).

The optimistic type of hope (A) is characterized by high levels of spiritual-religious (5.96), telic-temporal (5.89), and situational (5.43) hope, and a moderate level of affective hope (4.24). Moderate hope (B) is characterized by three mean results of a moderate value: in the spiritual-religious (4.71), affective (4.32), and telic-temporal (4.28) dimensions, and a low level of situational hope (3.74).

Patients with religious hope (C) exhibits more diversification of hope intensity in each of its dimensions, showing a very low level of situational hope (2.82), a low level of affective hope (3.90), a moderate level of telic-temporal hope (4.66), and a very high level of spiritual-religious hope (6.07).

The patients with weak hope (D) show a very low level of situational hope (2.25) and telic-temporal hope (2.88), and a low level of spiritual-religious hope (3.27). Only in the affective dimension do the patients exhibit hope of a moderate level (4.06).

As Figure 2 shows, for each type of cluster, the affective dimension of hope shows the lowest diversification of results. For the spiritual-religious dimension of hope, the mean values are close to clusters 1 and 2. Even though the localization of global mean values for clusters 2 and 3 is similar, the profile for cluster 2 is shallow, while cluster 3 shows larger differences between the mean values.

**Figure 3:**
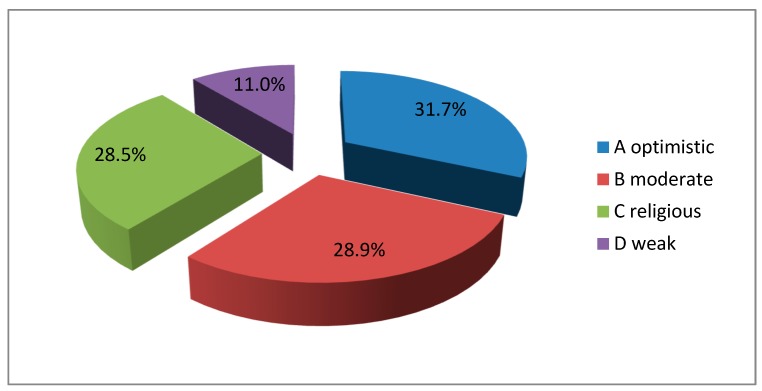
The percentage value of hope intensity for in the terminal phase of cancer.

According to Figure 3, the largest group was formed by patients with the optimistic type of hope (31.7%). Almost equally numerous groups were formed by the patients with moderate (28.9%) and religious (28.5%) types of hope. Only 11.0% of the patients were characterized by the weak type of hope.

**Figure 4:**
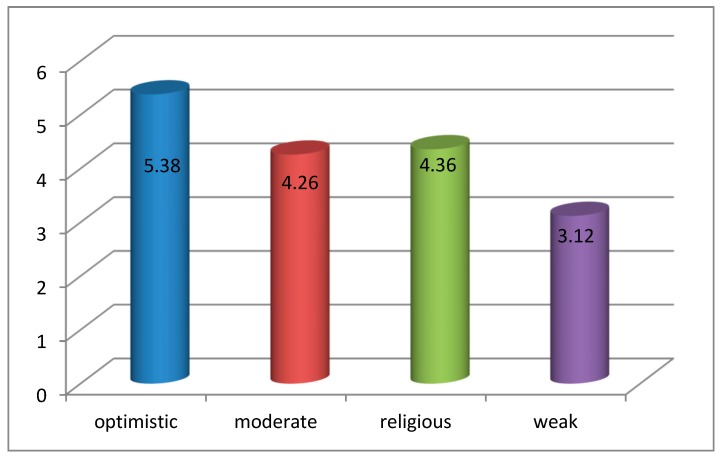
Intensity of particular types of hope in patients in the terminal phase of cancer.

The data presented in Figure 4 shows that the highest level of hope is observed in the patients with the optimistic type of hope, while the patients with weak hope show the lowest level of hope. The differences between the mean values are statistically significant (F = 280.636; *p* = 0.000).

## 4. Discussion

The development of science, medical advancement, and technological development all lead to therapeutic success. In the case of cancer, despite oncologic development, a full recovery can only be achieved for some patients [24]. Some others, after a long period of remission, face the terminal phase of cancer. This phase is a time of specialized care and treatment provided by the doctors. It is a time when the patients’ situations change and their future is uncertain. All this leaves a mark on the patients’ mental condition and the structure of their hope.

The first Polish study on hope in patients with cancer was performed by Block in 2010 [25]. The study showed that the patients had a strong level of hope (M = 5.25). The dominating component of hope was its telic-temporal (M = 5.79) and spiritual dimensions (M = 5.79), with a moderate level of hope in the affective (M = 4.55) and situational (M = 4.88) dimensions.

Our study shows that the patients in the terminal phase of cancer can be characterized by a low–moderate level of hope (M = 4.52). The profile of hope is dominated by its spiritual-religious component, where the level of hope is high (M = 5.34). In the telic-temporal dimension, the level of hope is moderate (M = 4.74), similarly to the affective dimension (M = 4.15). The internal structure of hope in patients with a serious, life-threatening disease and a poor prognosis for recovery offers the lowest value for the situational component (M = 3.85), which translates to the patients having little hope for recovery.

It should be concluded that the patients showed not only lower global results of hope, but also lower results for particular components of hope, with a similar hope profile. The differences may stem from the fact that the studied groups were questioned during different stages of cancer treatment. The study performed in 2010 measured hope in patients suffering from cancer for the first time in their lives who were subject to oncological treatment [25]. The study was performed in various health centers. The presented results concerned the patients in the terminal phase of cancer who were hospitalized in palliative and hospice wards. From a medical point of view, the chances for recovery equal zero, and the treatment is focused only on cancer symptoms. This may be related to diminished values of global hope, and its particular dimensions. The spiritual-religious dimension of hope remains high, even when the chances for recovery go down. The perspective of life being shortened leads to an increase in the telic-temporal level of hope.

More than half of the patients experienced global hope at a moderate level (50.81%), and a quarter of the patients at a low level, a very low level, or were devoid of hope (21.55%). In total, 25.20% of the patients had a high level of global hope.

The majority of the patients experience a very high or high level of hope in the spirituals-religious dimension (68.70%). They count on God for recovery in the difficult situation they find themselves. A high or very high level of hope in the telic-temporal dimension was experienced by 42.68% patients. More than a half of the patients experienced a moderate level of hope in the affective dimension. In the situational dimension, 55.18% of the patients experienced a low or very low level of hope, or no hope at all. These patients do not believe in therapy effectiveness or recovery.

The study from 2010 made a distinction between four types of hope: optimistic hope (A), hope of achieving desired goals (B), religious hope (C), and weak hope (D) [25]. 

The cluster analysis shows that there are four different types of hope, with a high statistical significance of difference (*p* ≤ 0.000) in the patients in the terminal phase of cancer. Given the level of hope in its particular dimensions, they can be classified as optimistic hope (A), moderate hope (B), religious hope (C), weak hope (D). Hope of achieving desired goals, which was distinguished in the study from 2010, was not observed in our study. This type of hope was characterized in the telic-temporal dimension by an orientation towards the future and a desire to achieve one’s goals (M = 6.2). In the situational dimension, it was characterized by a wish for recovery (M = 5.27). Examples of such goals can be to educate children, supervise their growth, participate in their wedding, or to see grandchildren. They give meaning to the efforts given in the battle against the disease.

Our classification considers moderate hope (B), which is an intermediary between A and D. It is characterized by the three mean results of a moderate value in the spiritual-religious, affective, and telic-temporal dimensions, and a low value in the situational dimension. For this type of hope, the lowest diversity of the mean results is observed for its particular components (0.97, while for A it is 1.72, for C it is 3.25, and for D it is 1.81).

The obtained results are similar to the ones from 2010 regarding the percentage of the patients in each hope group. The biggest group was the group with optimistic hope (33.33%), and the smallest group was the group with weak hope (14.29%).

In our study, the biggest group was formed by the patients with optimistic hope (31.7%). The groups with moderate hope (28.9%) and religious hope (28.5%) were almost equal. In total, 11.0% of the patients were categorized as weak hope patients. The highest values of hope were observed in the patients with optimistic hope, while the lowest values were observed in the patients with weak hope. There is a need to perform further studies in terminally ill groups of patients characterized by weak hope.

The phenomenon of optimistic hope being observed in the majority of the patients in the terminal phase of the disease may be related to Weinstein’s idea [26] of unrealistic optimism, which is conducive to seeing one’s future in a positive way. It is expressed through a reduction of negative emotional states connected with potential risks [27,28]. The correlation between unrealistic optimism with a will to live and to survive is also underscored as the most important aspect of mental well-being [29].

## 5. Conclusions

- The patients in the terminal phase of cancer experienced a moderate level of hope.
- The most intense hope was observed in the spiritual-religious dimension and the least intensive hope concerned chances of recovery.
- The patients showed varied levels of hope and varied internal structure of hope. More than half of them showed a moderate level of hope, and one-quarter of them showed a high level of hope.
- There were four types of hope observed across the patient groups: optimistic, moderate, religious, and weak.
- The biggest group of patients in the terminal phase of cancer was characterized by an optimistic type of hope, while the smallest group of patients was characterized by a weak type of hope.

## Figures and Tables

**Table 1:** The intensity of hope in hospitalized patients with cancer in a terminal phase. Descriptive statistics (*n* = 246).

Dimensions of Hope	Min	Max	M	Me	Mo	Number of Mode	SD
situational hope	1.13	7.00	3.85	3.63	3.50	14	1.37
telic-temporal hope	1.00	7.00	4.74	4.75	multiple value	15	1.20
spiritual-religious hope	1.00	7.00	5.34	5.50	5.13	18	1.16
affective hope	2.63	6.13	4.15	4.13	4.50	26	0.62
global hope	1.84	6.34	4.52	4.47	multiple value	7	0.79
ANOVA Friedman test, Chi2 ANOVA (*n* = 246, *df* = 3) = 225.3452 *p* = 0.00000							

Note: M = arithmetical mean; Me = median; Mo = modal; SD = standard deviation; ANOVA = analysis of variance.

**Table 2:** Results for numerous analyses of hope (*n* = 246).

No	Results Range	Intensity of Hope	Global Hope		Situational Hope		Telic-Temporal Hope		Spiritual-Religious Hope		Affective Hope	
			*n*	%	*n*	%	*n*	%	*n*	%	*n*	%
●	1–1.99	hopeless	1	0.41	17	6.91	5	2.03	2	0.81	-	-
●	2–2.99	very low	7	2.85	52	21.14	16	6.50	10	4.07	5	2.03
●	3–3.99	low	45	18.29	67	27.14	31	12.60	17	6.91	87	35.37
●	4–4.99	moderate	125	50.81	50	20.33	89	36.18	48	19.51	134	54.47
●	5–5.99	high	62	25.20	38	15.45	63	25.61	85	34.55	18	7.32
●	6–7.00	very high	6	2.44	22	8.94	42	17.07	84	34.15	2	0.81
Total			246	100.00	246	100.00	246	100.00	246	100.00	246	100.00

**Table 3:** Types of hope in patients in the terminal phase of cancer, differentiated by means of the cluster analysis, and significance of difference between the types.

Dimensions of Hope	Hope								Variance Analysis	
	Optimistic A		Moderate B		Religious C		Weak D			
	M	SD	M	SD	M	SD	M	SD	F	p
Situational	5.43	0.74	3.74	0.67	2.82	0.63	2.25	0.80	234.321	0.000
Telic-temporal	5.89	0.66	4.28	0.61	4.66	0.87	2.88	1.03	122.357	0.000
Spiritual-religious	5.96	0.69	4.71	0.69	6.07	0.60	3.27	0.98	141.319	0.000
Affective	4.24	0.64	4.32	0.56	3.90	0.57	4.06	0.63	6.763	0.000
Intensity of hope	5.38	0.39	4.26	0.31	4.36	0.38	3.12	0.45	280,636	0.000
